# Evolution of Pristine Emulsions and Hypothesis Explaining Their Existence

**DOI:** 10.3390/ijms27041837

**Published:** 2026-02-14

**Authors:** Andrei Dukhin, Renliang Xu, Darrell Velegol

**Affiliations:** 1 Dispersion Technology Inc., 364 Adams Street, Bedford Hills, NY 10507, USA; 2 Independent Researcher, 13084 NW 13 Street, Pembroke Pines, FL 33028, USA; renliang.xu@yahoo.com; 3 Department of Chemical Engineering, Penn State University, CBEB 225, University Park, PA 16802, USA; velegol@psu.edu

**Keywords:** pristine emulsion, droplet size, zeta potential, dielectrostatic force, surface tension, hydrophobic interaction

## Abstract

The term “pristine emulsion” is used for differentiating emulsions that consist of only water and oil with no surfactant from the Pickering emulsions, which are also surfactant-free but stabilized with colloidal particles. We review 22 papers dedicated to such emulsions prepared from a wide variety of liquids. We studied here the evolution of one such emulsion, hexadecane-in-water at 4% vl, over a long period of time, from days to weeks. We discovered that the droplet size grows with time, with a rate that depends on mixing conditions, which supports a coalescence hypothesis. However, this coalescence is unusual because the size reaches a certain constant value, which contradicts typical coalescence behavior. To explain this peculiarity and such emulsification in general, we employ a theoretical model that was developed for explaining pristine nano-bubble stability. We hypothesize the existence of a layer of structured water molecules at the interface, following Eastoe and Ellis (*Adv in Colloid and Interface Sci., 134–135, 89–95, 2007*) and others. We point out that the Electric Double Layer exerts a force on the water dipole moments in this layer (dielectrostatic force) that compensates Kelvin’s pressure. The droplet size calculated using this model is close to the measured size. The second factor associated with this layer is the repulsion of the water dipole moments, which we show can compensate for the surface tension tangential to the interface. After ruling out alternative hypotheses with our data, we conclude that the model suggested for explaining the stability of nano-bubbles is also consistent with our results for these “pristine emulsions”.

## 1. Introduction

There is an unusual type of emulsion described by several independent groups from different countries during the last 25 years [1,2,3,4,5,6,7,8,9,10,11,12,13,14,15,16,17,18,19,20,21,22]. These emulsions consist of only two liquids with no added surfactant. They are different from Pickering emulsions [23], which are stabilized with colloid particles. There is a term, “surfactant-free emulsions”, that is applied for classifying both of them, which can create confusion. This type of emulsion does not have any added surface stabilizing agent, in contrast to Pickering emulsions. Perhaps this was a motivation for Beattie and Djerdjev to introduce a special term for such a liquid–liquid interface—“pristine” [10]. It would be logical to call such emulsions “pristine emulsions” to distinguish them from Pickering emulsions [23].

Pristine emulsions are apparently very promising for controlling chemical kinetics, according to the extensive review by LaCour et al. [19]. This review describes examples of many organic and redox reactions that occur with much faster kinetics in water microdroplets and oil−water emulsions than in bulk solution.

We have summarized all published papers that we knew or could find on this subject in Table 1, listing all liquids used for making such emulsions. Authors of the cited papers express consensus in the long-term stability of these emulsions, lasting days and weeks. Table 1 lists all liquids used for making such emulsions. The list is quite diverse, which allows us to hypothesize that there is a common mechanism leading to the stability of these emulsions. One might assume that the lifespan of pristine emulsions would be very short. They would be destroyed either by coalescence, or by Oswald ripening [24], or both. Surprisingly, they turn out to be much more stable, with the emulsions demonstrating long-term stability lasting for days and even weeks.

The first hypothesis for explaining this paradoxical longevity would be the presence of impurities in the oil phase. Such impurities could serve as surfactants, reducing surface tension and thus promoting stability. There have been publications along these lines for both nano-bubbles and pristine emulsions. However, detailed verification experiments conducted by many groups with very thorough purification confirmed that longevity remains independently of the degree of cleaning. We provide several more arguments against this hypothesis based on results of this study in Appendix A. Nevertheless, even a small amount of impurities could affect the kinetics of coalescence, which should be taken into account for future verification tests.

A second hypothesis is that an electric surface charge at the pristine interface contributes to the observed stability. There is seemingly consensus on this. However, there is still some uncertainty regarding the origin of the surface charge. Most of the authors point toward adsorption of OH^−^ ions as the major factor, with vast supporting evidence collected mostly for degassed systems. There is another possibility in aqueous samples that are open to the atmosphere and consequently absorb CO_2_. The ionic composition of such systems is affected by the presence of bicarbonate anions, HCO_3_^−^, and carbonate anions, CO_3_^−2^, as shown, for instance, by Ninham and Nostro by measuring conductivity and phase separation [25]. Such open-to-air pristine emulsions could be considered as aqueous carbonated systems [26,27] with carbonic acid ions adsorbed at the interface. There are some serious arguments supporting this viewpoint presented in the papers by Ganachaud et al. [20,21,22]. We encountered some facts related to evolution of pH and ζ-potential that support this hypothesis as well. These facts are described in the full report of our study [28].

No matter what ions determine the droplet charge of pristine emulsions, their existence and longevity are paradoxical. There are many statements in the papers cited above declaring such paradoxical longevity, but there is not sufficient data for quantifying the statements. That is the main subject of this paper.

We study here the long-term longevity of hexadecane-in-water, 4% vl pristine emulsions with the pH adjusted to the basic range between 9 and 10. This pH adjustment was applied following the study by Djerdjev and Beattie, who showed that this range corresponds to the highest ζ potential [10]. In contrast to previous studies, we investigated the evolution of this emulsion over time by measuring the gradually growing droplet size continuously over days and even weeks. We discovered that the rate of this evolution depends on mixing conditions, which point to coalescence as the driving mechanism. Ostwald ripening is too slow to be the mechanism, according to our calculations based on the Lifshitz–Slyozov–Wagner (LSW) [29,30] theory (see Appendix B).

However, this coalescence is unusual because the emulsion droplet size seems to reach a constant submicron value. The explanation of this peculiarity is a key goal of this paper. Our explanation is based on the similarity between the paradoxical longevity of pristine emulsions and nano-bubbles [31,32]. There is a theoretical model that was developed for nano-bubbles, based on the assumption that there is a layer of structured water at the droplet interface [32]. A similar assumption was employed by many others, and there are recent experimental facts supporting this assumption, as we describe in the relevant papers in the Discussion section. We apply the same model here for pristine emulsions, and even further develop it in the theoretical section in Discussion below. This model predicts a constant droplet size that is quite close to the measured value.

## 2. Experimental Results

**Figure 1:**
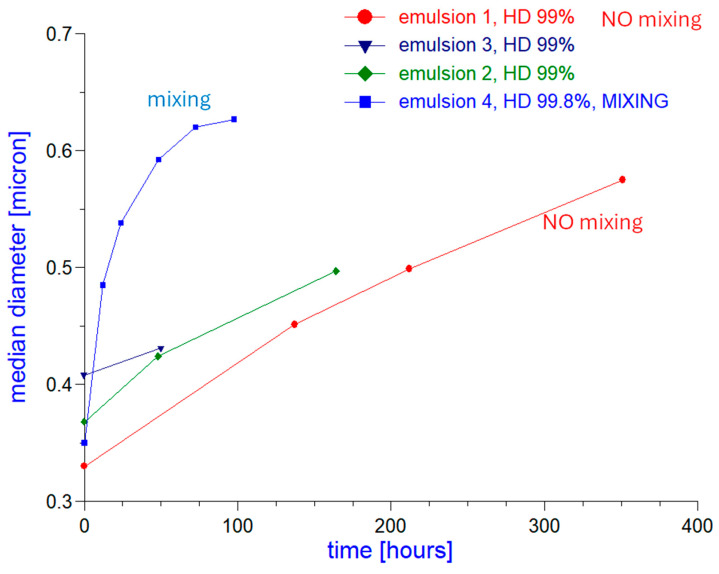
Median diameter measured for all four emulsions over time.

The evolution of the median droplet diameter is shown for all four emulsion samples in Figure 1. We have fewer points for emulsions 1, 2 and 3, but over a longer time than for emulsion 4, which was continuously mixed in contrary to the previous three. Emulsions 1, 2 and 3 were not mixed between measurements, they were stored in separate vials. 

It is seen that all these droplet size-time dependences merge towards the same value around 650 nm. The long-term evolution progress affects only the droplet size. The zeta potential remains constant after the initial 3 days of the emulsion’s preparation, as shown in Table 1 for emulsions 3 and 4.

The zeta potential values remain unchanged during emulsion evolution, as shown in Table 2. 

The conductivity of these emulsions was 0.032 ± 0.007 S/m, averaged over 24 measurements for 6 different loadings for all studied emulsions.

## 3. Discussion

The long-term evolution of the droplet size shown in Figure 1 points toward the existence of a stationary droplet size with a value around 650 nm. We propose using the theoretical model developed for bare (pristine) nano-bubbles, which also predicts the existence of the stationary nano-bubble size [32].

This model employs the assumption of the existence of a structured water layer at the interface with oriented water molecule dipoles. In fact, this idea is very old. According to Lyklema [33], the first mention of the water structure in the EDL was made by Hardy in 1915 [34], then Frank and Evans in 1945 [35], and Bockris in 1963 [36]. It was experimentally verified using atomic force microscopy by Israelachvili, Pashley, Ninham and Craig [37,38,39], theoretically supported by Derjaguin et al. [40] and Mansui-Ruckenstein [41], and mentioned in the books by Lyklema [33] and Hunter [42]. Recent studies of the dynamic surface tension of water [43], Raman spectroscopy of interfacial water layers [17], as well as surface charge [44] and electrophoretic mobility [45] measurements and water adsorption in nanopores measured with ultrasound [46] also indicate peculiarities in the water surface layer structure.

This hypothesis was suggested specifically for explaining the existence of pristine emulsions by Eastoe and Ellis [47]. Similar suggestion of the structured water interfacial layer affecting the interaction of bubbles was introduced by Craig, Ninham and Pashley [48].

However, neither the structured water layer nor the electric surface charge could explain the constant droplet size when taken separately. It is known from the Onsager–Samars theory [49] that the EDL causes only a few percent change in the surface tension. On the other hand, the interaction of the oriented water dipoles with the inhomogeneous electric field of the EDL offers such an explanation [32]. This interaction leads to two additional surface forces, one in the normal direction towards the interface and one in the tangential direction. We present theoretical models of these forces below.

### 3.1. Dielectrostatic Force Determines Stable Size

The first force is normal to the interface. Its origin is the gradient of the inhomogeneous electric field in the EDL acting on the dipole moments of the water molecules in the structured layer. It is known that such a force induces motion of objects that possess a dipole moment—dielectrophoretic motion [50]. In this case, the water molecules do not move, which suggests the term “dielectrostatic” for this force. It was shown in Ref. [32] that such a force could compensate completely for the excessive pressure in nano-bubbles. The balance of these two forces (i.e., the pressure force and the dielectrostatic force) leads to a stable nano-bubble size.

In the case of emulsion droplets, the dielectrostatic force competes with the excess pressure caused by the interfacial curvature—Kelvin’s effect [51]. We hypothesize that this balance also leads to the observed stable droplet size of our studied emulsions at the end of long-term evolution.

We can test this hypothesis by calculating the radius of such stable droplets (a_stable_) using the following Equation derived in Ref. [32]:

(1) $$a_{stable}=\frac{2\gamma }{\zeta \times \kappa^{2}\times d_{w}\times L_{str}\times N_{A}\times cw}\;$$

where γ = 0.055 [N/m] is the surface tension of the hexadecane–water interface, d_w_ = 6.17 × 10^−30^ [C-m] is the dipole moment of a water molecule, N_A_ = 6.02 × 10^23^ [1/mol] is Avogadro’s number, Cw = 55,000 mol/m^3^ for water, L_str_ is the thickness of the structured water layer, ζ is the zeta potential, and 1/κ is the Debye length.

The last three parameters bring some uncertainty into the theory.

The first is the thickness of the structured water layer. We assume two monolayers of water molecules, which makes L_str_ = 0.48 × 10^−9^ m. This number was used and justified by Mansui and Ruckentein [41] in their theory of electrokinetics with a structured water layer.

The second one is the Debye length. The ionic composition of the emulsion is too complex for using the classical Debye formula as a sum of ionic contributions [52]. The list of ions includes ions of carbonic acid dissociations associated with CO_2_ adsorption, HCO_3_^−^ and CO_3_^−2^, in addition to Na^+^, Cl^−^, H^+^, and OH^−^. Therefore, the most appropriate way for estimating Debye length is based on using conductivity as described in the ISO Standard [53] and the Ref. [54]. This Dukhin–Goetz method is based on the equation that was derived by comparing two different definitions for the critical frequency of Maxwell–Wagner relaxation:

(2) $$\kappa^{-1}\approx \sqrt{\frac{\varepsilon_{m}\varepsilon_{0}D_{eff}}{K_{m}}}$$

where ε_o_ is the dielectric permittivity of vacuum (8.85 × 10^−12^ F/m), ε_m_ is the dielectric constant of water (78), *K_m_* is the measured conductivity (0.032 S/m), and *D_eff_* is the effective diffusion coefficient. This equation yields the following equation for *κ*^2^:

(3) $$\kappa^{2}=\frac{K_{m}}{\varepsilon_{0}\;\varepsilon_{m}\;D_{eff}}\;$$

The main uncertainty in this equation comes from the unknown effective diffusion coefficient *D_eff_*. However, this parameter varies over a limited range. The diffusion coefficients of most ions in aqueous solutions, except H^+^ and OH^−^, are in the range between 0.6 and 2 in 10^−9^ m^2^/s. Here are the diffusion coefficients of the ions: Na^+^—1.33 × 10^−9^ m^2^/s, Cl^−^—2.0 × 10^−9^ m^2^/s, HCO_3_^−^—1.18 × 10^−9^ m^2^/s, CO_3_^−2^—0.955 × 10^−9^ m^2^/s.

We assume that the effective coefficient *D_eff_* = 1.2 × 10^−9^ m^2^/s. This assumption is associated with a potential error of ~10–20%.

Substituting all parameter values into Equation (3) leads to the following values of *κ*^2^ and the Debye length *κ*^−1^:

(4) $$\kappa^{2}=\frac{0.032\;[\frac{S}{m}]}{8.85\times 10^{-12}\left[\frac{F}{m}\right]\times 78\times 1.2\times 10^{-9}[\frac{m^{2}}{sec}]}=3.86\times 10^{16}\;\left[\frac{1}{m^{2}}\right];\;\kappa^{-1}=5.1\;\left[nm\right]$$

The last uncertain parameter is the ζ potential. Table 2 reports values calculated using the Smoluchowski approximation [52] to be roughly −40 mV on average. This model ignores the effect of the surface conductivity. The software of the instrument that we used has an option of advanced electrokinetic theory described in the ISO Standard [53]. This procedure requires information on the parameter κ*a*, where *a* is the droplet radius. This parameter equals roughly 60, because κ^−1^ equals 5.1 nm and a stable droplet radius is roughly 300 nm. The high value of this parameter leads to rather small correction in the ζ-potential, only about 5 mV on average. Such corrected ζ-potential would become −45 mV.

There is one more potential correction that we must employ. Equation (1) contains the surface potential, not the ζ-potential. We used ζ-potential because surface potential is usually unknown. Here, we can attempt to calculate the surface potential, assuming that the slip plane associated with ζ-potential coincides with the external border of the structured water layer, at a distance 0.48 nm from the interface. For conducting such recalculation, we can employ the equation suggested by Hunter [42]:

(5) $$EXP\left(-\kappa L_{str}\right)=\frac{tanh\frac{F\zeta }{4RT}}{tanh\frac{F\psi }{4RT}}\;$$

where ψ is the surface potential, F is the Faraday constant, R is the gas constant, and T is absolute temperature. This correction elevates the |ζ|-potential to 50.2 mV.

Substituting these parameters into Equation (1) leads to the following theoretical value of the stable droplet size:

(6) $${d_{stable}=2\times a}_{stable}=\frac{2\times 2\times 0.055}{\;50\times 10^{-3}\times 3.86\times 10^{16}\times 6.17\times 10^{-30}\times 0.48\times 10^{-9}\times 6.02\times 10^{23}\times 55,500}\;\approx 1.1\;micron$$

This value is about 30% higher than the experimentally observed one. There is one more correction that could potentially reduce it—surface conductivity through the Stern layer. It amplifies the calculated surface potential. If we assume it as −80 mV, then the theoretical and experimental values would agree.

This is supportive of the hypothesis that the balance of normal forces between excess Kelvin’s pressure and dielectrostatic force might indeed control the droplet size.

There is one more aspect associated with the dielectrostatic force. Introduction of this force disrupts the balance between Kelvin’s pressure and the surface tension. There must be other factors that compete with the surface tension at the stationary state. Repulsion of the water molecule dipole moments in the structured water interfacial layer could play this role. We suggest some calculation of the magnitude of this factor in the next section.

### 3.2. Repulsion of Oriented Water Dipoles

The aligned dipole moments of the parallel-oriented water molecules in the structured surface layer repel each other, contributing to the lateral interactions at the interface. We can characterize its contribution to the surface tension and assign the symbol γ_str_. We will try to estimate the value of this contribution and compare it with the known experimental value of water–hexadecane surface tension, which is 0.055 N/m.

We begin with a general definition of the surface tension γ that can be found in Lyklema’s book [33],

(7) $$\gamma ={\left(\frac{\partial U}{\partial A}\right)}_{V}$$

where U is energy, and A is surface area.

Let us assume that one water molecule with dipole moment d_w_ comes into this layer. It would cause a change in the energy by ΔU_dd_ due to interacting with other molecules’ dipole moments and increase the surface by ΔA. Therefore, the contribution to the surface tension approximately equals:

(8) $$\gamma_{str}=\;\frac{∆U_{dd}}{∆A}$$

**Figure 2:**
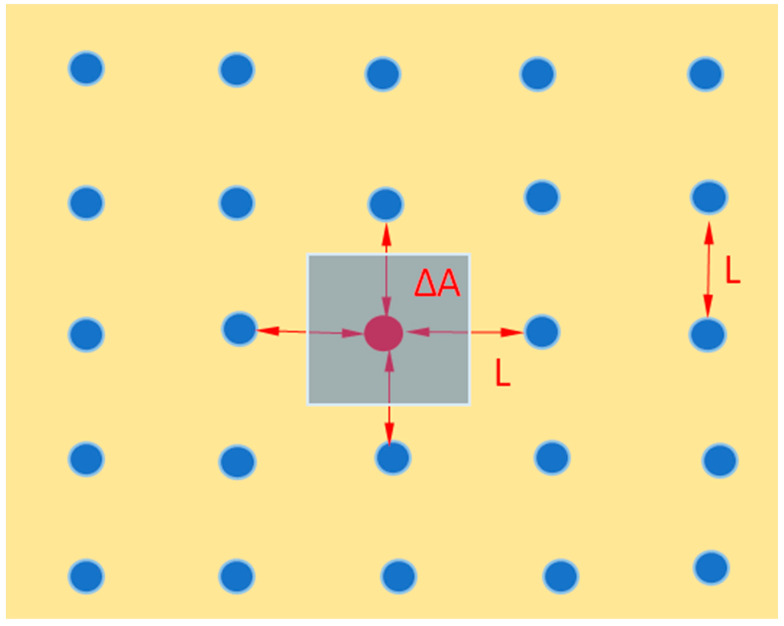
A region of the water–oil interface with blue circles symbolizing water molecules oriented perpendicularly to the interface (i.e., into the page). The red circle in the center is the imaginary added molecule. The shaded area around it illustrates ΔA.

In order to estimate the energy of the dipole–dipole interaction, we consider interactions only with the molecular nearest neighbors of the added molecule. Figure 2 illustrates the simplest symmetrical positioning of water molecules in the element of the interfacial layer. We are using a simple square lattice and considering only the nearest four neighbors. Our calculation here is to check if the repulsion resulting from the parallel dipoles is at all comparable to the surface tension. The distance between molecules (L) is taken as constant. It is seen that the added molecule has roughly four neighboring molecules.

We also adopt a simple additive approach to estimate the interaction energy.

The interaction energy U_dd_ of two parallel dipoles d_w_ in a medium having dielectric constant ε is:

(9) $$U_{dd}=\;\frac{d_{w}^{2}}{2\pi \varepsilon \varepsilon_{0}r^{3}}$$

where ε_0_ is dielectric permittivity of vacuum, ε is the relative permittivity of the fluid, and r is distance between centers of the dipoles.

Assuming that the added molecule interacts with four others as shown in Figure 2, the total change in surface energy due to this dipole–dipole interaction equals:

(10) $${∆U}_{dd}=\;4\times \frac{d_{w}^{2}}{2\pi \varepsilon \varepsilon_{0}L^{3}}$$

where we use r = L, according to Figure 2.

The change in the surface area ΔA equals:

(11) $$∆A=\;L^{2}$$

Substituting Equations (10) and (11) into Equation (8) leads to Equation (12):

(12) $$\gamma_{str}=\frac{2\times d_{w}^{2}}{\pi \varepsilon \varepsilon_{0}L^{5}}$$

The average distance between water molecules can be estimated from the fact that 1 m^3^ of water contains 55,500 moles [(1000 kg/0.018 kg/mol)]. The volume (L^3^) corresponding to the single molecule equals 1 m^3^ divided by 55,500 × N_A_. We find L from Equation (13):

(13) $$L=\sqrt[{3}]{\frac{1}{55,500\times 6.2*10^{23}}}\;\approx 0.299\;nm$$

Now, we can estimate the value of γ_str_ using the values of all parameters from the papers [55,56,57,58,59,60]:

(14) $$\gamma_{str}=\frac{2\times d_{w}^{2}}{\pi \varepsilon \varepsilon_{0\;}L^{5}}=\frac{2\times 6.17^{2}\times 10^{-60}}{3.14\times 80\times 8.85\times 10^{-12}\times {\left(0.3\right)}^{5}\times 10^{-45}}=\frac{2\times 6.17\times 6.17}{3.14\times 8\times 8.83\times 3^{5}\times 0.1}\approx 0.014\;N/m$$

It turns out that the potential contribution of oriented water dipoles’ repulsion to the surface tension can be close to the known value of the hexadecane–water interface—0.055 N/m. It can be even closer because this calculation underestimates the dipole–dipole repulsion.We assumed the distance between them in the surface layer was the same as in the bulk—0.3 nm. However, it should be smaller because of surface tension pushing them closer. A reduction in this distance even by a small amount would have a large impact on their repulsion due to the fifth power dependence. It turns out that there would be complete compensation of the two effects if the average distance between water dipoles in the structured layer reduced to 0.23 nm.

### 3.3. Balance of Forces at Pristine Interfaces with Structured Water Layer

It is usually assumed that only two forces are acting at the water–oil interface of an emulsion droplet: surface tension and the Kelvin pressure due to curvature.

**Figure 3:**
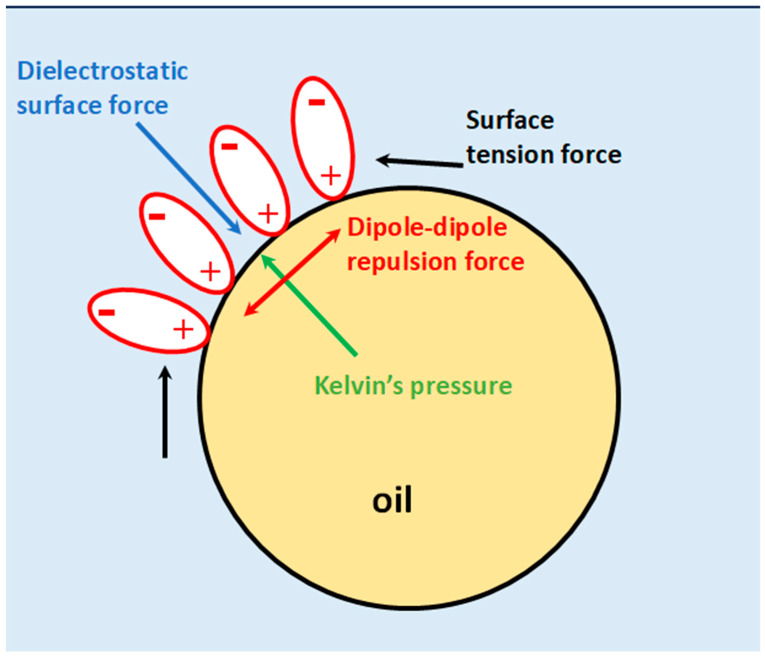
Illustration of the force balance at a pristine water–oil interface with a structured layer of water molecules. Usually only two forces (Kelvin pressure and surface tension) are considered; however, we are hypothesizing the existence of two additional forces not previously recognized (the inward dielectrostatic force and repulsive dipole–dipole force).

The existence of the EDL at the interface is an additional factor. However, it is not sufficient by itself to explain our experimental observation. That is why we employ the hypothesis of the structured water layer. This hypothesis leads to two more forces that counteract with the classical ones. This new force balance is shown in Figure 3.

There are two normal forces: the Kelvin pressure and dielectrostatic force. They completely compensate each other at a particular droplet size, given with Equation (1). There are two lateral forces at a given local position at the interface: surface tension and repulsion of aligned dipoles of the water molecules.

### 3.4. Liquids That Could Form “Pristine Emulsions”

Not all liquids can form pristine emulsion. We tried, for instance, toluene, and it failed. It is possible that the capability of creating such pristine emulsion is linked to the small size of the toluene molecule. Larger molecules can have a more stable emulsion. The molecular weight of toluene 92 g/mol is much smaller than the molecular weight of hexadecane 226 g/mol.

There is an insightful analysis of the water molecule’s structure building by non-polar molecules given by Frank and Evans [35]. They attributed insolubility of non-polar substances in water more to the entropy effect rather than to the internal energy. They wrote: …“*Large, non-polar molecules have stronger van der Waals force fields around them than do small ones, and are more strongly held in any condensed phase, including aqueous solutions. The larger they are, however, the larger the iceberg which they produce in water, and therefore the greater the loss in entropy involved in dissolving them…*”.

Future studies would reveal if such a simple explanation is valid.

## 4. Methods, Materials, and Instruments

We conducted an experiment with the hexadecane-in-water emulsion similar to Beattie and Djerdjev [10]. Their method of developing pristine emulsion contains a lot of chemical purification procedures and prevention of CO_2_ adsorption by using an N_2_ atmosphere. The motivation of such an approach is clear—elimination of impurities as possible surfactants. However, it creates an impression that pristine emulsions are a rare and exotic fluke. The authors of that paper wrote that they were able to replicate their results under conventional conditions when samples were open to air. That is what we wanted to test for removing a veil of mystery from pristine emulsions.

Therefore, we initially used hexadecane (HD) from Sigma-Aldrich (St. Louis, MO, USA) with purity specified as ≥99%. We prepared several emulsions using this HD. We report the results of three of them, labeled as emulsions 1, 2, and 3. Then, after discussing results with several experts in the field, we were criticized that this HD is not sufficiently pure. Reference to the detailed Beattie and Djerdjev work [9] was not sufficient. Thus, we obtained cleaner HD from Sigma-Aldrich with purity specified as ≥99.8%, which was purified with all available methods by the producer The potential amount of impurities is 5 times smaller. We calculated coverage of the droplet interfaces with these possible 0.2% impurities. These calculations reveal that it is not sufficient for surface coverage. 

For water, we used store-bought distilled water.

The water ionic strength was adjusted to 0.001 M using NaCl from Sigma-Aldrich.

The pH was adjusted using a 0.1 N solution of NaOH.

For preparing the initial sample mixture, we added 3.7 g of hexadecane to 114.9 g of 0.001 M NaCl aqueous solution with a high pH. This would ensure 4% vl emulsions, assuming that we could mix these liquids. The initial pH value was close to 9 for emulsions 1, 2, and 3 and very close to 10 for emulsion 4. Emulsions 1, 2, and 3 were prepared in separate glass vials. Emulsion 4 was prepared directly in the measurement cell of the measuring instrument.

We sonicated the mixture for 1 min using a high-power 20 KHz, 100-watt horn sonic probe by Sonic & Materials Inc. (Newtown, CT, USA) since it is known that sonication causes emulsification [61,62]. This method allows manual mixing of the sample and brought the top layers of the liquid into the sonic jet stream. This indeed leads to the formation of an opaque liquid with no visible phase border line. The pH value of the mixture drops, reflecting adsorption of OH^−^ ions. Then, we re-adjust the pH back to the initial value (9–10 range) by adding small amounts of NaOH, and we sonicate again for 1 min. Emulsions must be mixed by magnetic mixing or pumping to ensure the homogeneous spreading of NaOH.

After that, we let the emulsion evolve for up to 24 h, keeping it mixed with a low-power agitator, using a magnetic mixer. We repeat such cycles 3 times over 3 days, which allowed us to achieve the smallest droplet size of about 350 nm. Further sonication and equilibration were not effective in reducing the droplet size.

It turns out that the emulsion undergoes an evolution of the droplet size after these initial 3 days. For monitoring this long-term evolution, we saved emulsions 1, 2, and 3 in closed vials and measured them in intervals of 5 days. Emulsions 1, 2, and 3 stayed idle in the glass vial between measurements. They were mixed, but not sonicated, only during measurement when poured back into the measuring cell every 5 days. In contrast, emulsion 4 remained in the measuring cell and underwent continuous mixing and measurement of both the zeta potential and droplet size distribution. However, we kept measuring this emulsion for two more days, just 5 measurements per day for monitoring the long-term evolution of droplet size. Then, we stopped because the droplet size reached a constant value.

We used the Acoustic and Electroacoustic Spectrometer DT-1202, by Dispersion Technology Inc., Bedford Hills, NY, USA. This instrument has an acoustic sensor for measuring the ultrasound attenuation spectra within the frequency range from 3 MHz to 100 MHz. These spectra are the raw data used for calculating the droplet size distribution.

This instrument also has an electroacoustic zeta potential probe that measures the Colloid Vibration Current (CVI), which is the raw data for calculating the droplet zeta potential. All details can be found in Ref. [54] and the ISO standards [53,63,64,65]. This instrument can also measure conductivity, pH, and temperature.

The functionality and calibration of the zeta potential probe of DT-1202 was verified by measuring the zeta potential of silica Ludox certified reference material [66] before and after every experiment.

This instrument has a built-in magnetic mixer that creates extra pressure on the bottom of the cell, which in turn pumps liquid through the cell. It is possible to adjust the rate of pumping by setting the magnetic cross rotation to different speeds.

## 5. Conclusions

There have been multiple studies indicating that certain liquids form stable oil-in-water and water-in-oil emulsion with a longevity on the scale of days, even weeks, without surfactants or any other surface-stabilizing substance. We adopt the term “pristine emulsion” for these mixtures following Beattie and Djerdjev [10]. We reproduced emulsions from that study (4% vl hexadecane-in-water) by adjusting the water pH above 9 and then applying sonication in several consecutive steps. We modified the Beattie and Djerdjev approach somewhat. Instead of de-gassing it, we did not isolate the emulsion from CO_2_, which leads to the formation of carbonic acid in the water phase. We achieved full emulsification when all added hexadecane formed droplets. Such an emulsion remains relatively stable for hundreds of hours, but droplet properties exhibit an evolution. The growth rate of the droplet size depends on the mixing conditions, which suggests coalescence as a driving mechanism. There is one peculiarity of this coalescence—the droplet size reaches a constant value. This cannot be explained by the existence of the electric surface charge, which does not explain the emulsification process because it does not ensure sufficient reduction in the surface tension. That is why we employ the hypothesis of the structured water layer at the interface, as was suggested for such emulsions previously by Eastoe and Ellis [47] and for bubbles by Craig, Ninham and Pashley [38].

The structured water layer by itself is also insufficient for explaining the observed long-term evolution patterns. However, the interaction of the EDL with the structured water layer offers such an explanation. It was shown in Ref. [32] for nano-bubbles, another object with a “pristine interface”, that the interaction of these two layers leads to two new surface forces: dielectrostatic that acts in the normal direction and repulsion of aligned water dipoles acting in the tangential direction. We suggest extending this model to pristine emulsions.

The dielectrostatic force could balance Kelvin’s pressure force, which can lead to the observed stable droplet size of the emulsion after a long-term evolution. The calculated droplet size turns out to be somewhat close to the experimentally measured droplet size. The lateral force of repulsion between oriented water dipoles in the structed layer compensates for surface tension.

## Figures and Tables

**Table 1:** Summary of publications on “pristine emulsions”. All emulsions consist of water and oil. The oil phase might be different. We list these oils in the third column, which is labeled “oil”.

Year	Country (and Affiliation) of Authors [Reference]	Oil
**Oil-in-Water Emulsions**		
1999–2018	Japan [1,2,3,4,5,6]	Hexadecane Hexane Benzene Oleic acid Esters of oleic acid
2003–2004	University of California, USA [7,8]	Dodecane Hexane Octane Decane Octadecane Squalane Hexamethylsqualane 4-fluorotoluene
2004	Bristol University, UK [9]	Dodecane
2004, 2025	Beattie and Djerdjev from Sydney University, Australia [10,11]	Hexadecane Decane Dodecane Eicosane Squalane Perfluoromethyl decalin
2020	China [17]	Hexadecane
2022	Korea [12]	Olive oil
2023	China, Japan, Canada [16]	Decane
2025	USA and China [18]	Hexadecane
2018, 2025	France [20,21,22]	Hexadecane
**Water-in-oil emulsion**		
2010–2018	Japan [13,14,15]	Cyclohexane Dodecane Benzene Octane Hexane Oleic acid

**Table 2:** Values of zeta potential for emulsions 3 and 4, organized as experiments consisting of several consecutive measurements. Emulsion 3 was stored in a closed vial, which was shaken before the sample was poured into the measuring cell. Emulsion 4 was continuously mixed inside the measuring cell even after initial steps of preparation.

Emulsion 3			Emulsion 4		
Experiment Date	Measurement Date	Zeta [mV]	Experiment Date	Measurement Date	Zeta [mV]
19 September 2024 14:36	19 September 2024 14:38	−40.8	24 November 2024 13:14	24 November 2024 13:25	−34.3
	19 September 2024 14:41	−40.4		24 November 2024 13:32	−34.5
	19 September 2024 14:43	−40.8		24 November 2024 13:39	−34.8
19 September 2024 15:22	19 September 2024 15:26	−35.5		24 November 2024 13:48	−34.2
	19 September 2024 15:28	−34.2		24 November 2024 13:55	−34.3
	19 September 2024 15:31	−35.2	25 November 2024 12:29	25 November 2024 12:39	−36.3
	19 September 2024 15:33	−34.6		25 November 2024 12:47	−35.7
23 September 2024 9:43	23 September 2024 9:46	−42.2		25 November 2024 12:55	−35.4
	23 September 2024 9:48	−40.8		25 November 2024 13:03	−35.6
	23 September 2024 9:50	−40.4		25 November 2024 13:12	−35.4
	23 September 2024 9:53	−40.6	26 November 2024 13:41	26 November 2024 13:52	−35.1
	23 September 2024 9:55	−43.3		26 November 2024 13:59	−35.2
26 September 2024 10:31	26 September 2024 10:44	−43.6		26 November 2024 14:06	−35.4
	26 September 2024 10:54	−38.4		26 November 2024 14:13	−35.1
	26 September 2024 11:04	−43.9		26 November 2024 14:22	−34.9
	26 September 2024 11:13	−43.2	27 November 2024 11:41	27 November 2024 11:52	−36.2
	26 September 2024 11:22	−38.3		27 November 2024 11:59	−36.4
				27 November 2024 12:07	−35.9

## Data Availability

There are some data at the archive https://arxiv.org/pdf/2509.13529, ref. [28]. Detail database of all experimental results could be requested from the Dispersion Technology Inc CEO—A.D.

## Appendix A. Arguments Against the Hypothesis of Impurities as the Origin of the Observed Emulsification

First, several authors specifically mentioned that high reproducibility of their experiments ruled out impurities as potential explanation of emulsification.

Second, we also tested this hypothesis by multiple sonication steps. If surfactant-like impurities are responsible for the observed emulsion stability, then a simple one-step sonication would produce such emulsions. Instead, we observed that multiple sonication steps with pH adjustments and equilibration are required. We describe the full procedure in the section on Materials and Sample preparation. A similar preparation procedure was described by Beattie and Djerdiev [10]. The impurities hypothesis cannot explain why these multiple steps are required for preparing pristine emulsions.

Third, we conducted our experiments using hexadecane following the method of Beattie and Djerdjev [10], with >99.8% pure material from Sigma-Aldrich. Here, we present calculations indicating that 0.2% of surface-active impurity cannot provide sufficient surface coverage for emulsions with submicron droplet size. For all these reasons, we rule out the surfactant impurities hypothesis.

Let us calculate the amount of surfactant for complete coverage of a spherical droplet with radius a_drop_. We assume that surfactant molecules can be characterized approximately with radius a_surf_. Then, the required volume fraction of surfactant φ_cr_ equals to the ratio of the spherical layer around the droplet with thickness 2 × a_surf_ to the droplet volume:

(A1) $$\varphi_{cr}=\frac{\left(4\pi a_{drop}^{2}\;2a_{surf}\right)}{\frac{4\;}{3}\;\pi a_{drop}^{3}}=\frac{6a_{surf}}{a_{drop}}$$

We have determined that the radius of the stable droplet size is about 350 nm, according to Figure 1.

For estimating the radius of the potential surface-active molecules that could be present in the hexadecane samples as impurities, we assume that the impurities should have a molecular weight similar to hexadecane. Otherwise, it would be eliminated by the purification procedure. There is information about the molecular size of substances with a molecular weight that is close to the molecular weight of hexadecane (229) in the paper [55]. The radius of glucose molecules with a molecular weight of 180 is 0.33 nm, whereas the radius of sucrose molecules with a molecular weight of 342 is 0.44 nm. Therefore, we can assume that the radius of any potential surface-active impurity a_surf_ is about 0.4 nm. Substituting these numbers into Equation (A2) yields the following:

(A2) $$\varphi_{cr}=\frac{6a_{surf}}{a_{drop}}=\frac{6\times 0.4}{350}\;=0.007=0.7\%$$

The cleanest hexadecane that we use contains less than 0.2% of impurities. This means that in the worst case, when all impurities are surface-active, they would cover only 30% of the droplet interface. This number overestimates the surface coverage because in reality, not all impurities would be located at the interface. A significant part would stay in the droplet bulk for maintaining surface–bulk equilibrium. Therefore, impurities could not stabilize the interface and explain the observed effects.

However, small amounts of impurities could affect coalescence kinetics. This might lead to a variation of results from one emulsion sample to another.

## Appendix B. Calculation of the Lifshitz–Slyozov–Wagner (LSW) [29,30] Time for the Kinetics of Ostwald Ripening [24]

Ostwald ripening is a phenomenon observed in emulsions that involves the change of a droplet size over time due to transfer of emulsion droplet liquid from small droplets into the larger ones. This is a thermodynamically driven process caused by excessive pressure in the emulsion droplets due to curvature of their interfaces. It is usually referred to as Kelvin’s pressure [51]. This excess pressure amplifies the chemical potential of the molecules of the liquid that forms the emulsion droplets. It becomes higher in smaller droplets compared to larger droplets. The resulting diffusion flux moves these molecules from small droplets to larger ones. This description stresses the importance of polydispersity for Ostwald ripening [24]. However, it is possible to derive an equation for the evolution of an average particle volume, assuming some typical droplet size distribution. This was done by Lifshitz and Slyozov [29,30], who derived an equation for characterizing the kinetics of Ostwald ripening:

(A3) $$\frac{da^{3}}{dt}=\frac{8DC_{\infty }\gamma M}{9\rho^{2}RT}$$

Definitions of all symbols in this equation are:

- a is an average radius of the droplets in [m].
- t is time in [s].
- D is the diffusion coefficient of the emulsion droplet liquid molecules in [m^2^/s].
- C_sol_ is solubility in [g/m^3^].
- γ is the surface tension in [N/m].
- M is the molecular weight in [g/mol].
- ρ is the density in [g/m^3^].
- R is the gas constant in [J/mol-K].
- T is the absolute temperature in K.

This theory yields the following equation predicting the value of the droplet radius at the time moment *t*, due to Ostwald ripening:

(A4) $$a\left(t\right)=a_{0}\sqrt[{3}]{1+t\frac{8DC_{sol}\gamma M}{9\rho^{2}RTa_{0}^{3}}}=a_{0}\sqrt[{3}]{1+\frac{t}{t_{lsw}}}$$

where a_0_ is an average droplet radius of the initial emulsion, at *t* = 0.

Parameter t_lsw_ equals:

(A5) $$t_{lsw}=\frac{9\rho^{2}RTa_{0}^{3}}{8DC_{sol}\gamma M}$$

This parameter t_lsw_ (LSW time) can be considered as the characteristic time of Ostwald ripening, because an average droplet volume doubles during this time, according to Equation (A4).

We can calculate the LSW time for a 4%vl hexadecane-in-water emulsion, finding values of all required parameters of hexadecane in the literature [55,56,57,58,59,60]. We assume the value of the initial droplet radius to be 0.2 micron, or 0.2 × 10^−6^ m. The result is 38 days. This is much longer than the duration of our experiments. Therefore, we can conclude that Ostwald ripening does not affect the observed emulsion evolution.
